# Contrast Enhancement Method Using Region-Based Dynamic Clipping Technique for LWIR-Based Thermal Camera of Night Vision Systems

**DOI:** 10.3390/s24123829

**Published:** 2024-06-13

**Authors:** Cheol-Ho Choi, Joonhwan Han, Jeongwoo Cha, Hyunmin Choi, Jungho Shin, Taehyun Kim, Hyun Woo Oh

**Affiliations:** Pangyo R&D Center, Hanwha Systems Co., Ltd., 188, Pangyoyeok-ro, Bundang-gu, Sengnam-si 13524, Gyeonggi-do, Republic of Korea; joonhwan.han@hanwha.com (J.H.); jeongwoo.cha@hanwha.com (J.C.); hyunmin.choi@hanwha.com (H.C.); jh.hoya.shin@hanwha.com (J.S.); taetae@hanwha.com (T.K.); hyunwoo.oh@hanwha.com (H.W.O.)

**Keywords:** infrared, thermal image, image processing, histogram equalization, contrast enhancement, night vision

## Abstract

In the autonomous driving industry, there is a growing trend to employ long-wave infrared (LWIR)-based uncooled thermal-imaging cameras, capable of robustly collecting data even in extreme environments. Consequently, both industry and academia are actively researching contrast-enhancement techniques to improve the quality of LWIR-based thermal-imaging cameras. However, most research results only showcase experimental outcomes using mass-produced products that already incorporate contrast-enhancement techniques. Put differently, there is a lack of experimental data on contrast enhancement post-non-uniformity (NUC) and temperature compensation (TC) processes, which generate the images seen in the final products. To bridge this gap, we propose a histogram equalization (HE)-based contrast enhancement method that incorporates a region-based clipping technique. Furthermore, we present experimental results on the images obtained after applying NUC and TC processes. We simultaneously conducted visual and qualitative performance evaluations on images acquired after NUC and TC processes. In the visual evaluation, it was confirmed that the proposed method improves image clarity and contrast ratio compared to conventional HE-based methods, even in challenging driving scenarios such as tunnels. In the qualitative evaluation, the proposed method demonstrated upper-middle-class rankings in both image quality and processing speed metrics. Therefore, our proposed method proves to be effective for the essential contrast enhancement process in LWIR-based uncooled thermal-imaging cameras intended for autonomous driving platforms.

## 1. Introduction

Autonomous driving platforms relying on cameras, such as visible light-based RGB (red-green-blue) or YUV with CMOS (complementary metal oxide semiconductor) technology, exhibit remarkable performance in object detection, recognition, and information provision [1,2]. However, a significant drawback arises in nighttime environments where obtaining high-quality image data for object recognition becomes challenging due to the absence of ambient light [3].

To address this limitation in the automotive and defense industries, ongoing research is actively exploring the integration of night vision systems using infrared cameras. Infrared-based commercial cameras utilize the infrared wavelength range and are generally divided into three product groups that utilize specific wavelength ranges: (1) SWIR (short-wave infrared, 0.9–1.7 μm), (2) MWIR (mid-wave infrared, 3–5 μm), and (3) LWIR (long-wave infrared, 8–14 μm) [4,5,6].

In SWIR-based cameras, the principle is that when energy from the light source hits an object and reflects, the detector visualizes the reflected energy. Therefore, in places such as underground parking lots or tunnels where there is no light, SWIR-based cameras have the disadvantage of not being able to obtain valuable images that users can utilize. For this reason, the size of the product increases because a light source is essential to operate under various conditions and a cooled detector must be used. The need to use a cooled detector increases the price of the product and requires significant power consumption due to the larger size of the products. Consequently, SWIR-based cameras are generally used mainly in the defense industry.

MWIR- and LWIR-based cameras are generally known to users as thermal-imaging cameras. MWIR-based cameras can acquire information on objects located at a distance because their atmospheric transmittance is relatively high compared to cameras that utilize other infrared wavelengths. However, like SWIR-based cameras, they must utilize a cooled detector, which increases the size of the product and the cost of production, and requires a large amount of power consumption. On the other hand, LWIR-based cameras have the advantage of being able to acquire information across a wide temperature range because they can detect most of the thermal energy emitted by various targets. Additionally, LWIR-based cameras can use bolometer-type detectors, allowing the use of either a cooled or uncooled detector depending on the intended use of the product. This means that products can be manufactured with characteristics such as low power consumption, low cost, and miniaturization, depending on the intended use.

Although LWIR-based cameras are known to have relatively shorter detection ranges than MWIR-based cameras, they provide performance that satisfies most distance conditions for situational awareness according to various standards (e.g., ISO-26262 [7]) or user requirements [4]. Therefore, to meet various standards or requirements such as cost or other conditions, depending on the application, LWIR-based cameras can be utilized most widely.

For this reason, ongoing academic research is actively exploring the integration of night vision systems using LWIR-based thermal-imaging cameras into autonomous vehicle platforms [8,9,10]. Especially in the case of research and development (R&D) centers within the automotive industry, LWIR-based cameras are being developed for use as night vision systems among various infrared wavelengths, as they must produce finished products that meet standards such as ISO-26262. LWIR-based thermal-imaging cameras typically employ two detectors, categorized as either (1) cooled or (2) uncooled [11,12,13]. Cooled detectors provide high-quality image acquisition but are expensive to produce, large in size, and require significant power, so they are mainly used in applications such as defense. On the other hand, uncooled detectors, which are cheaper to produce, smaller in size, and require less power, are preferred in autonomous vehicle platforms within the automotive industry. However, since uncooled detectors do not have a mechanical cooler, noise removal and pixel value correction for temperature changes require additional processing of the raw data collected.

Essential pre-processing steps, such as non-uniformity correction (NUC) to address fixed pattern noise and temperature compensation (TC) to offset temperature-related pixel value variations, are crucial for resolving hardware-related issues [14]. Nevertheless, images obtained after NUC and TC processes exhibit low-dynamic range (LDR) characteristics, rendering them unsuitable for deep learning or machine learning-based object detection and recognition, essential components in autonomous vehicle platforms. To overcome this challenge, research is underway to develop contrast-enhancement techniques, specifically aiming to convert LDR into high dynamic range (HDR) characteristic images after NUC and TC processes.

Various histogram equalization (HE)-based methods exist for image contrast enhancement. Most commercially available products commonly utilize global histogram equalization (HE)-based methods after non-uniformity correction (NUC) and temperature compensation (TC) processes to enhance image quality. This approach helps reduce production costs and ensures stability, meeting military standards (MilSpecs) or international standards organization (ISO)-26262 requirements in autonomous or military industries. However, conventional HE methods, relying on a probability density function (PDF) and cumulative distribution function (CDF), can oversaturate results when histogram values are excessively concentrated, leading to issues such as a shifted average brightness level. Moreover, conventional HE-based methods solely present performance evaluations on data obtained from mass-produced products. This means there is a lack of experimental results on contrast enhancement using images calculated exclusively with NUC and TC processes. Additionally, most studies employing these methods only conduct experiments in driving scenes characterized by good image quality and favorable driving scenarios. Consequently, it becomes challenging to assert the algorithm suitability for infrared thermal-imaging cameras in autonomous driving platforms since the performance evaluation is confined to specific favorable driving conditions. Therefore, to comprehensively assess performance for deployment in autonomous driving platforms, it is imperative to conduct experiments in worst-case driving environments, including scenarios such as tunnels.

In this paper, we introduce a four-group-based HE method designed for contrast enhancement. Additionally, we present experimental results demonstrating the effectiveness of contrast enhancement using images after NUC and TC processes, considering both best and worst driving scenarios. The primary objective of our proposed method is to exhibit contrast enhancement performance in both favorable and challenging driving conditions. Moreover, regarding the experimental results, the comparison between the proposed method and conventional methods in both best and worst driving scenarios enables a comprehensive evaluation. In conclusion, the obtained images serve to determine the most suitable contrast-enhancement technique after the NUC and TC processes, providing potential application probability in mass-produced products.

## 2. Background

### 2.1. Non-Uniformity Correction (NUC)

All multidimensional array sensors generate a fixed pattern due to geometric differences between each pixel element or gain difference in the transmission and amplification stages, known as fixed pattern noise (FPN). Specifically, when using uncooled infrared detectors designed by a read-out integrated circuit (ROIC) that processes row and column units, FPN manifests as line patterns in both horizontal and vertical directions, referred to as non-uniformity in the infrared image. To address this issue, the method for correcting non-uniformity is known as NUC in infrared-based thermal imaging systems. In embedded environments, a two-reference NUC is commonly employed, utilizing Equation (1), with two reference input values required for this operation.

(1) $$I_{N}\left(x,y\right)=G_{N}\left(x,y\right)\times I\left(x,y\right)+O_{N}\left(x,y\right)$$

where $G_{N}\left(x,y\right)$ represents the look-up table (LUT) of gain for the NUC, I(x,y) denotes the input image, $O_{N}\left(x,y\right)$ is the LUT of offset for NUC, and $I_{N}\left(x,y\right)$ corresponds to the output image after the NUC operation.

### 2.2. Temperature Compensation (TC)

The process of adjusting the output value in response to temperature changes in an uncooled infrared detector is commonly referred to as TC or the thermal electric cooler (TEC)-less algorithm. This is necessary because the pixel values, influenced by temperature in the output of an uncooled infrared detector without TEC, need to be compensated to a constant value. In the absence of TEC, the image output values for the same object may vary inconsistently due to changes in the external/internal environment, potentially leading to fixed patterns caused by noise and temperature fluctuations. To address this, after collecting image data for a specific temperature using a black body, pixel values corresponding to each temperature, which vary based on the internal/external environment, are stored and applied in real time for each temperature. The TC method achieves this objective by incorporating an offset value, as illustrated in Equation (2).

(2) $$I_{T}\left(x,y\right)=I\left(x,y\right)-O_{T}\left(x,y\right)$$

where $I_{T}\left(x,y\right)$ denotes the output image after applying the TC operation, I(x,y) represents the input image, and $O_{T}\left(x,y\right)$ is the LUT of offset for TC.

There are two options for implementing NUC and TC operations as software in an embedded environment. When TC is performed after NUC, Equation (3) is applicable. Conversely, when NUC is performed after TC, it is equivalent to Equation (4).

(3) $$I_{O}\left(x,y\right)=G_{N}\left(x,y\right)\times \left(I\left(x,y\right)-O_{T}\left(x,y\right)\right)+O_{N}\left(x,y\right)$$

(4) $$I_{O}\left(x,y\right)=G_{N}\left(x,y\right)\times I\left(x,y\right)+\left(O_{N}\left(x,y\right)-O_{T}\left(x,y\right)\right)$$

where $I_{O}\left(x,y\right)$ represents the output image obtained by applying both NUC and TC. Currently, there is no quantitative numerical report that definitively determines the superiority of either Equation (3) or Equation (4). However, in general, the implementation and research are trending towards performing TC after NUC, as shown in Equation (3).

### 2.3. Contrast Enhancement (CE)

The difference in image output values between objects in the output image after NUC and TC processing is minimal, making it challenging to distinguish the disparities. When image data values are concentrated at a specific pixel point, the resulting image exhibits a poor contrast ratio. Hence, there is a need to enhance contrast by evenly rearranging the distribution of slightly different image data values.

For this purpose, HE-based methods are widely employed in embedded environments. This is particularly relevant because the mass production cost of the embedded thermal imaging system is high, and high performance processors cannot be utilized. In essence, HE-based methods are chosen due to challenges such as processor occupancy resulting from NUC and TC processes.

In HE methods, two types are commonly distinguished: (1) Global and (2) Local. After using NUC and TC processes, pixel values tend to concentrate in a specific pixel intensity region. Consequently, when window- or cell-based local HE is applied after NUC and TC, the contrast improvement rate may be very low. Therefore, it is essential to perform global HE after NUC and TC processes, with most mass-produced thermal imaging systems adopting global HE as the subsequent step following NUC with TC.

In the contrast enhancement methods after the proposed 2011 year, as shown in Table 1, it is typically divided into three types except for deep learning-based methods as shown in Table 1: (1) histogram-based, (2) retinex-based, and (3) other technique-based. In histogram-based contrast enhancement methods, there are various methods, and reflectance-guided contrast accumulated histogram equalization (RG-CACHE) was proposed as one of the state-of-the-art methods. In retinex-based contrast enhancement methods, low-light image enhancement via illumination map estimation (LIME) is widely used because of its high contrast improvement performance. In other technique-based contrast enhancement methods, there are various methods, and their algorithms utilize de-haze or statistical techniques. Among these three types of contrast enhancement methods, the retinex-based contrast enhancement method showed higher contrast enhancement performance than the other two types of contrast enhancement methods for general visible images, equivalent to RGB or YUV cameras. However, retinex-based contrast enhancement methods have a complex computation process. On the other hand, the other two types of contrast enhancement methods showed relatively lower contrast improvement performance than the retinex-based contrast enhancement method. However, these methods have the advantage of a simple computation process compared to retinex-based contrast enhancement methods.

## 3. Proposed Method

When using NUC and TC, as detailed in Equations (1)–(4) and mentioned in Section 2 contrast enhancement is essential to provide meaningful images to users. Pixel values processed through NUC and TC are in a 14-bit format. If a contrast enhancement algorithm is performed using a high-performance processor in an embedded environment, the 14-bit image data produced through the NUC and TC algorithms can be utilized.

However, as mentioned in Section 1, to produce a finished product suitable for the automotive industry, components must be standard specification such as ISO-26262. In other words, to produce an LWIR-based camera for automotive, processors and memories must comply with ISO-26262 standard specifications. Additionally, Original Equipment Manufacturers (OEMs) require products like cameras to have low-power and low-production cost characteristics.

For LWIR-based camera products to satisfy OEM requirements, they must use low-cost and low-power memory (e.g., LPDDR2 or LPDDR3) and low-cost processors (e.g., TI TDA3x) while meeting the ISO-26262 standard. In such as embedded platform environment, to ensure acceptable image quality for OEMs and other users, communication with various external components (e.g., Controller Area Network (CAN) or I^2^C) and cyber-security must be possible. Additionally, NUC, TC, global contrast enhancement, and local contrast enhancement algorithms are all operational.

Therefore, when both global and local contrast enhancement algorithms are utilized, the 14-bit format data obtained after performing NUC and TC are generally reduced to 8-bit. This reduction is necessary to meet various conditions such as processing speed and power consumption. Considering that a local contrast enhancement algorithm will be included in the future, this paper proposes a global contrast enhancement algorithm based on 8-bit image data.

### 3.1. Motivation

When acquiring an image in 14-bit format after performing NUC and TC operations, it is inevitable that pixel values are concentrated in a specific area due to the characteristics of the LWIR-based camera. Therefore, when downscaling the image data from 14-bit to 8-bit (after applying the automatic gain control described later), the low-temperature, medium-temperature including room-temperature, and high-temperature areas can be more clearly distinguished. As a result, the temperature areas are more distinct in the 8-bit domain (space) than in the 14-bit domain (space).

**Figure 1:**
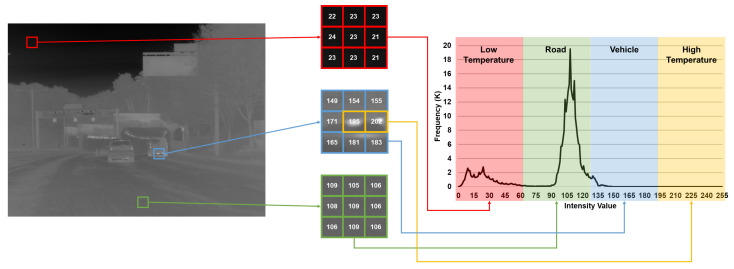
Pixel value analysis result of downscaled image by using NUC and TC methods.

From this perspective, the analysis results shown in Figure 1 were confirmed. Figure 1 presents the results of analyzing the values of each pixel after downscaling the image, calculated using NUC and TC, to 8-bit format. As shown in Figure 1, in a typical driving scenario with NUC and TC, the pixel values of the infrared-based thermal images fall within the low-temperature, medium-temperature, and high-temperature ranges in the histogram plot (low-temperature range: 0 to 63, medium-temperature range: 64 to 191, and high-temperature range: 192 to 255).

The red annotation area in the thermal image represents the sky and the pixel values were confirmed to be in the early 20s. In the case of the green annotation located on the asphalt road, it was observed that the pixel values were in the early 100s. Lastly, in the case of the blue annotation located on the rear side of the vehicle, it can be observed that the pixel values of the vehicle fall within the range of 149 to 183. However, the exhaust pipe of the vehicle has a slightly higher value of 195 or higher than the surrounding objects due to the high temperature.

When viewed as a histogram plot, pixel values can be grouped into a total of four regions. First, in the case of the sky with a lower temperature compared to surrounding objects (without the sun), it falls within the first group (region) with pixel values between 0 and 63. Second, on vehicle-driving roads such as asphalt, it falls within the second group (region) between 64 and 127. Third, the vehicle falls within the pixel group (region) between 128 and 191. Lastly, parts expressing high temperatures, such as exhaust pipe or the sun, belong to the fourth group (region) with a pixel value of 192 or higher. In other words, in terms of histogram frequencies, unlike CMOS-based cameras, it was observed that the pixel values from the 8-bit infrared thermal image exhibit the characteristic of being clustered in specific groups (regions).

### 3.2. Algorithm

**Figure 2:**
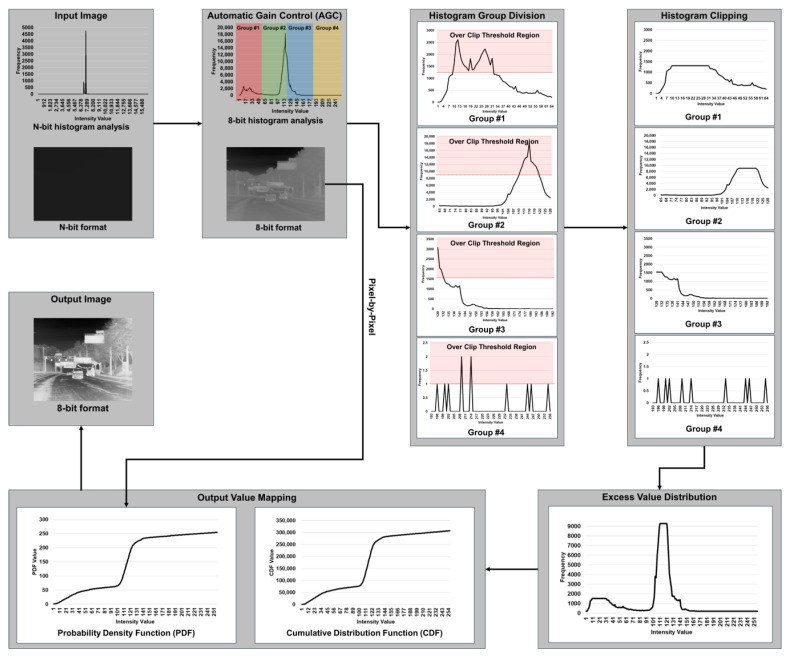
Operation process of the proposed method.

Based on the analysis results depicted in Figure 1, we propose a region-based HE method that utilizes clipping and distribution techniques with a dynamic clip limit. As illustrated in Figure 2 and pseudo-code (Algorithm 1) for the proposed method, the operational process of the proposed method comprises five steps: (1) Automatic Gain Control (AGC) including Histogram Bin Calculation, (2) Histogram Group Division, (3) Histogram Clipping, (4) Excess Value Distribution, and (5) Output Value Mapping.

#### 3.2.1. Automatic Gain Control (AGC) and Histogram Bin Calculation

In the AGC step, prior to Histogram Bin Calculation, the bit depth of the input pixel values in the computed image, obtained using NUC and TC methods, is reduced from an N-bit format to an 8-bit format, as defined by Equations (5)–(7). Thereafter, in the Histogram Bin Calculation step, the frequencies of pixel values across the entire image in 8-bit format are then computed.

(5) $$I_{max}=max\left(I_{NT}\right)$$

(6) $$I_{min}=min\left(I_{NT}\right)$$

(7) $$I_{\alpha }=\frac{\left(I_{NT}-I_{min}\right)}{\left(I_{max}-I_{min}\right)}\times 2^{\alpha }$$

where $I_{NT}$ represents the input image computed by using the NUC and TC methods, whereas $I_{max}$ and $I_{min}$ denote the maximum and minimum pixel values of $I_{NT}$, respectively. The variable α corresponds to the desired bit reduction from the input pixel value to the output pixel value, and α represents the output image with α-bit depth.
**Algorithm 1** Pseudo-Code for Proposed Contrast Enhancement Method Using Region-Based Histogram Equalization with Dynamic Clipping Technique**Input:** $I_{In}$: Input Image with *N*-bit format **Output:** $I_{\left(Out,L\right)}$: Contrast Enhanced Output Image
  1:**< Automatic Gain Control (AGC) >**  2:$I_{In.Max}$ ← max$\left(I_{In}\right)$  3:$I_{In.Min}$ ← min$\left(I_{In}\right)$  4:$I_{8bits}$ ← 255 × ($I_{In}-I_{In.Min}$) / ($I_{In.Max}-I_{In.Min}$)  5:   6:**< Histogram Bin Calculation >**  7:**for** x←1 to *N* **do**  8:   **for** y←1 to *M* **do**  9:     $H(I_{8bits})$ ← $H(I_{8bits})$ + 110:   **end for**11:**end for**12: 13:**< Histogram Group Division >**14:$H_{1}$ ← H(0:63)15:$H_{2}$ ← H(64:127)16:$H_{3}$ ← H(128:191)17:$H_{4}$ ← H(192:255)18: 19:**< Histogram Clipping >**20:**for** L←1 to 4 **do**21:   $H_{L.Max}$ ← max($H_{L}$)22:   $H_{L.Min}$ ← small($H_{L}$)23:   [$H_{L}$, $E_{L}$] ← Clip($H_{L}$, $H_{L.Max}$, $H_{L.Min}$)24:**end for**25: 36:**< Excess Value Distribution >**27:$H_{C}$ ← [$H_{1}$, $H_{2}$, $H_{3}$, $H_{4}$]28:*E* ← $E_{1}$ + $E_{2}$ + $E_{3}$ + $E_{4}$29:$H_{C}$ ← $H_{C}$ + (*E* / 256)30: 31:**< Output Value Mapping >**32:*C* ← CDF($H_{C}$)33:*P* ← PDF(*C*)34:**for** x←1 to *N* **do**35:   **for** y←1 to *M* **do**36:     O(x,y) ← 255 × $P(I_{8bits})$37:   **end for**38:**end for**

#### 3.2.2. Histogram Group Division

After computing the pixel value frequencies in the Histogram Bin Calculation step, the Histogram Group Division step involves dividing the histogram bin values into four groups, based on the analysis results presented in Figure 1 and defined by Equations (8) and (9).

(8) $$\left(i,k\right)=\left\{\begin{array}{cc} \left(1,n\right) & n\leq 63\\ \left(2,n-64\right) & n\leq 127\\ \left(3,n-128\right) & n\leq 191\\ \left(4,n-192\right) & other \end{array}\right.$$

(9) $$Hist_{i}\left(k\right)=Hist\left(n\right)$$

where *i* represents the histogram region with values ranging from 1 to 4, and *n* represents the histogram bin value. In the first histogram region, frequencies are considered for bin values ranging from 0 to 63. The second histogram region includes frequencies for bin values from 64 to 127, whereas the third histogram region encompasses frequencies for bin values from 128 to 191. Finally, the fourth region comprises frequencies for bin values from 192 to 255. In other words, each histogram region is subdivided into four, with each region having a histogram bin value range of 64.

As explained in the motivation subsection, each frame captured by the LWIR-based camera contains extensive temperature information. By applying AGC, the analysis can be performed within a limited domain (space), allowing the intervals to be distinguished based on the temperature range. For example, when the temperature decreases (e.g., from the sky to below zero), the pixel value approaches 0. Conversely, when the temperature increases (e.g., from a car engine or a fire), the pixel value approaches 255. In the case of medium temperature, the range is considerably wider. However, even within the intermediate temperature range, the thermal inversion phenomenon is generally observed based on the median value within the limited domain (space), depending on the external environment temperature (e.g., in the 8-bit domain, the pixel value is 127). Therefore, the intermediate temperature range is divided into two histogram regions. As a result, the proposed method divides the histogram into four regions, allowing the pixel values within each temperature region to be distinguished and utilized.

#### 3.2.3. Histogram Clipping

In the Histogram Clipping step, the frequency values of histogram bin values for each region are clipped using Equations (10)–(13).

(10) $$D_{Hist}\left(i\right)=max\left(Hist_{i}\left(k\right)\right)-small\left(Hist_{i}\left(k\right)\right)$$

(11) $$C_{T}=D_{Hist}\left(i\right)\times \alpha$$

(12) $$E_{i}=\left\{\begin{array}{cc} E_{i}=E_{i} & Hist_{i}\left(k\right)\leq C_{T}\\ E_{i}=E_{i}+\left(Hist_{i}\left(k\right)-C_{T}\right) & Hist_{i}\left(k\right)>C_{T} \end{array}\right.$$

(13) $$Hist_{i}\left(k\right)=\left\{\begin{array}{cc} Hist_{i}\left(k\right) & Hist_{i}\left(k\right)\leq C_{T}\\ C_{T} & Hist_{i}\left(k\right)>C_{T} \end{array}\right.$$

where *i* represents the histogram region; $max(Hist_{i}\left(k\right))$ is the maximum histogram frequency value of region *i*; $small(Hist_{i}\left(k\right))$ is the minimum histogram frequency value of group *i* (minimum non-zero value if a histogram exists, if the histogram does not exist in group *i*); $D_{Hist}\left(i\right)$ is the difference value between maximum and minimum histogram frequency values; α is the weight factor for selecting the threshold for the clipping operation; $E_{i}$ is the excess value for each region. As shown in Equations (10) and (11), the difference in frequency values between the maximum and minimum histogram frequencies is calculated for each region.

Subsequently, the threshold value for the clipping operation is determined using the difference value and the weight factor α, which ranges from 0 to 1.

After determining the threshold value, as shown in Equation (13), the histogram frequency values for each group are clipped. During the clipping operation, similar to the clip-limit adaptive HE (CLAHE) method [28], the excess value is calculated using Equation (12). When the histogram frequency value is greater than the threshold value for the clipping operation, the histogram frequency value is adjusted to the threshold value. Conversely, when the histogram frequency value is less than the threshold value, the histogram frequency value remains unchanged.

#### 3.2.4. Excess Value Distribution

In the Excess Value Distribution step, the excess value is distributed for each histogram region by using Equation (14).

(14) $$Hist_{i}\left(k\right)=Hist_{i}\left(k\right)+\frac{\sum_{i=1}^{4}E_{i}}{256}$$

As illustrated in Equation (14), first, the summed excess value is divided by 256, and then the resulting divided excess value is added to the clipped histogram frequency values from bin value 0 to 255. This distribution process, as per Equation (14), helps prevent oversaturation of histogram frequency values at specific points during CDF computation.

#### 3.2.5. Output Value Mapping

In the Output Value Mapping step, the output value is then calculated using Equations (15)–(18).

(15) $$V_{C}\left(\left(i-1\right)\times 63+k\right)=\sum_{i=1}^{4}\sum_{n=0}^{63}Hist_{i}\left(k\right)$$

(16) $$V_{C}\left(n\right)=\frac{V_{C}\left(n\right)}{\left(w\times h\right)}$$

(17) $$V_{S}=V_{C}\left(I_{\alpha }\left(x,y\right)\right)$$

(18) $$V_{O}\left(x,y\right)=\left\{\begin{array}{cc} V_{S}\times 255 & V_{s}\leq 1\\ 255 & V_{s}>1 \end{array}\right.$$

where $V_{C}\left(\left(i-1\right)\times 63+k\right)$ is the calculated value by using the cumulative distribution function (CDF); *w* and *h* are the width and height size of the computed image using the NUC and TC methods, as follow:

where $V_{C}\left(\left(i-1\right)\times 63+k\right)$ is the calculated CDF value; $V_{C}\left(n\right)$ is the normalized CDF by using *w* and *h*; *w* and *h* are width and height size of the computed image using the NUC and TC methods; $V_{S}$ is the selected CDF value using $V_{C}\left(n\right)$; and $V_{O}\left(x,y\right)$ is the output image with improved contrast ratio. Using Equations (15) and (16), the CDF value can be calculated. Subsequently, the CDF value is selected using the 8-bit computed input image obtained through the NUC and TC methods. Finally, normalization is performed, and the output value is calculated using the selected CDF value multiplied by 255. During the computation of the output value, it is fixed to 255 when the selected CDF value is greater than one, as the CDF value can exceed one due to the excess value distribution process.

## 4. Experimental Results

To evaluate the performance of the proposed method, it is crucial to set the parameter α for determining the threshold value for Histogram Clipping in each histogram region. In Section 4, α was set to 0.5. This choice was made because if α approaches 1, the Histogram Clipping result for each histogram region according to the threshold value becomes very weak, resulting in no significant difference from traditional histogram equalization (THE). Conversely, if α approaches 0, the Histogram Clipping result for each histogram region can have a very strong effect. However, as per Equations (14)–(18), when pixel values are densely distributed in a specific histogram region, the contrast enhancement performance can be significantly reduced as α approaches 0. Additionally, there is a risk that the average pixel level of the image after contrast enhancement processing may decrease, leading to a notable reduction in image brightness. Therefore, in this paper, the experimental results obtained by setting α to 0.5, which is the median value between 0 and 1, were compared with the other conventional methods.

### 4.1. Qualitative Comparison (Visual Comparison)

#### 4.1.1. Best Driving Scenario

**Figure 3:**
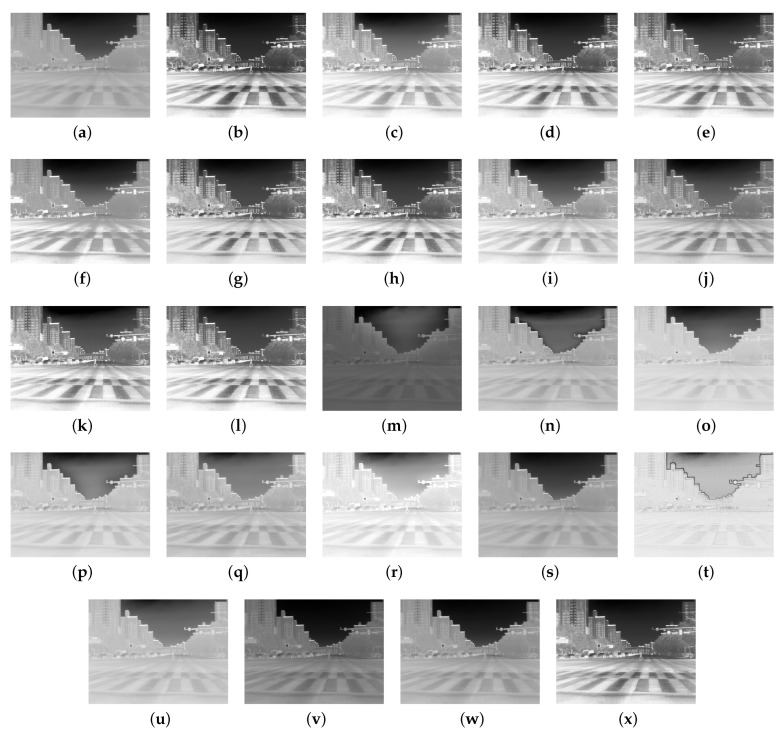
Experimental results obtained under the best driving scenario with vehicles on the road: (**a**) input image with 8-bit, (**b**) THE, (**c**) BBHE [29], (**d**) DSIHE [30], (**e**) MMBEBHE [31], (**f**) RMSHE [32], (**g**) BPDHE [33], (**h**) BPHEME [34], (**i**) BHEPL [35], (**j**) RLBHE [15], (**k**) RG-CACHE [16], (**l**) ROPE [17], (**m**) AMSR [18], (**n**) NPE [19], (**o**) SIRE [20], (**p**) SRIE [23], (**q**) MF [21], (**r**) LIME [24], (**s**) SRLLIE [22], (**t**) Dong [25], (**u**) MEFF [26], (**v**) Al-Ameen [27], (**w**) Al-Ameen [36], and (**x**) proposed method with 0.5 weight factor for clipping operation.

Figure 3 presents experimental results comparing the proposed method with various conventional contrast enhancement methods for visual comparison under the best driving scenario. First, Figure 3b–l shows the experimental results using histogram-based conventional contrast enhancement methods. Second, Figure 3m–r shows the experimental results using retinex-based conventional contrast enhancement methods. Third, Figure 3s–w shows the experimental results using other technique-based (e.g., de-haze) conventional contrast enhancement methods. Finally Figure 3x shows the experimental result using the proposed method based on histogram techniques. As depicted in Figure 3a, the downscaled 8-bit image obtained using NUC, TC, and AGC reveals objects such as crosswalks, vehicles, and apartments.

Among the histogram-based contrast enhancement methods, Figure 3c,f,i,j showcases the results when using BBHE (Brightness-Preserving Bi-Histogram Equalization) [29], RMSHE (Recursive Mean-Separate Histogram Equalization) [32], BHEPL (Bi-Histogram Equalization with a Plateau Limit) [35], and RLBHE (Range-Limited Bi-Histogram Equalization) [15]. These methods improve the contrast ratio compared to the input image, making detailed object components visible. However, the image clarity appears somewhat diminished, akin to a foggy appearance.

Conversely, Figure 3d,e,g displays the outcomes when utilizing DSIHE (Dualistic Sub-Image Histogram Equalization) [30], MMBEBHE (Minimum Mean Brightness Error Bi-Histogram Equalization) [31], and BPDHE (Brightness-Preserving Dynamic Histogram Equalization) [33]. These methods exhibit increased sharpness compared to BBHE [29], RMSHE [32], BHEPL [35], and RLBHE [15] results. However, oversaturation of pixel values is observed in trees and signs on the far left of the image, causing inaccuracies in object details and pixel values.

In contrast, Figure 3b,h,k,l,x demonstrates that applying THE, BPHEME (Brightness-Preserving Histogram Equalization with Maximum Entropy) [34], RG-CACHE [16], ROPE (Reflectance-Oriented Probabilistic Equalization) [17], and the proposed method yield better contrast enhancement performance in terms of contrast ratio and sharpness compared to conventional methods. Detailed parts of images enhanced using THE, BPHEME, RG-CACHE, ROPE, and the proposed method are clearly visible, surpassing the results obtained with other histogram-based conventional methods.

Among the retinex-based contrast enhancement methods, as shown in Figure 3m, AMSR (Adaptive Multi-Scale Retinex) [18] exhibited low pixel-level values and poor contrast enhancement performance. As depicted in Figure 3r, when using LIME [24], it showed an oversaturated experimental result compared to other conventional and proposed methods. On the other hand, as shown in Figure 3n–q,s, when using NPE (Naturalness Preserved Enhancement) [19], SIRE (Simultaneous Illumination and Reflectance Estimation) [20], SRIE (Simultaneous Reflectance and Illumination Estimation) [23], MF (Multi-Scale Fusion) [21], and SRLLIE (Structure-Revealing Low-Light Image Enhancement) [22], they exhibited better contrast enhancement performance than other retinex-based methods. However, generally, these methods showed relatively lower contrast enhancement performance compared to histogram-based contrast enhancement methods.

Among the other technique-based contrast enhancement methods, the method proposed by Dong [25] showed good edge enhancement in the image compared to other methods. However, in terms of contrast enhancement, the pixel values are oversaturated compared to other methods. In other words, the contrast enhancement performance is lower than other contrast enhancement methods. As shown in Figure 3u–w, they demonstrate better contrast-enhanced experimental results compared to the results obtained when using the method proposed by Dong [25]. However, they exhibit lower contrast enhancement performance compared to the experimental results using histogram-based contrast enhancement methods.

**Figure 4:**
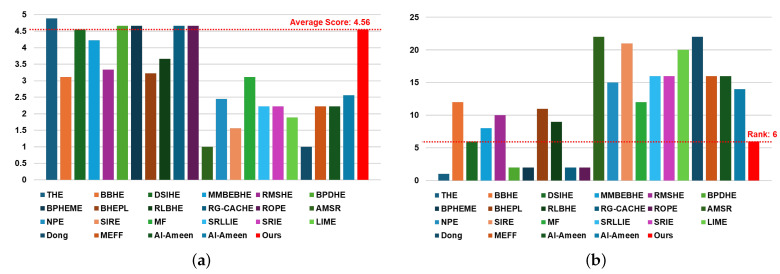
Chart to visually compare Table 2: (**a**) average score and (**b**) rank.

Table 2 and Figure 4 present the results of subjective evaluations based on blind tests of 5 min videos (equivalent to 300 frames) containing frames from the best driving scenario, conducted by nine individuals including R&D engineers working in the automotive or military industries. Table 2 displays three items: (1) subjective scores for each individual, (2) average score, and (3) rank; Figure 4 illustrates graphs containing two items: (1) average score and (2) rank.

Subjective evaluation scores for each individual range from one to five points, with one point indicating the video consisting of the worst quality frames and five points indicating the video consisting of the best quality frames. The average score is calculated by summing up the subjective scores evaluated for each method and dividing by the number of individuals (nine in Table 2). The rank value is determined by ranking the calculated average scores from top to bottom.

As evident in Table 2 and Figure 4, our method obtained an average score of 4.56 and ranked 6th when sorted from top to bottom. Being ranked 6th implies being within the top 30% (approximately within the 7th rank) overall. When analyzing solely within the histogram-based contrast enhancement method category and ranking sequentially, the proposed method is positioned in the 6th rank, indicating it received a medium average score. Compared to contrast enhancement methods based on retinex and other techniques, it is apparent that the proposed method achieves a better rank than conventional methods. In other words, through subjective evaluation, which visually assesses the image, it is confirmed that histogram-based contrast enhancement methods are most effective in the best driving scenario.

#### 4.1.2. Worst Driving Scenario

**Figure 5:**
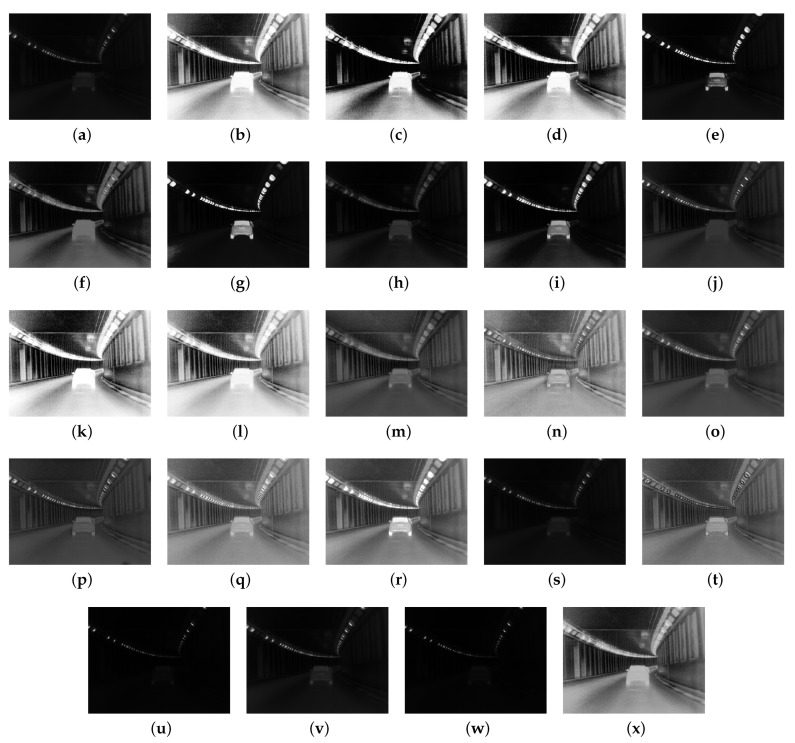
Experimental results obtained under the worst driving scenario located in the tunnel: (**a**) input image with 8-bit, (**b**) THE, (**c**) BBHE [29], (**d**) DSIHE [30], (**e**) MMBEBHE [31], (**f**) RMSHE [32], (**g**) BPDHE [33], (**h**) BPHEME [34], (**i**) BHEPL [35], (**j**) RLBHE [15], (**k**) RG-CACHE [16], (**l**) ROPE [17], (**m**) AMSR [18], (**n**) NPE [19], (**o**) SIRE [20], (**p**) SRIE [23], (**q**) MF [21], (**r**) LIME [24], (**s**) SRLLIE [22], (**t**) Dong [25], (**u**) MEFF [26], (**v**) Al-Ameen [27], (**w**) Al-Ameen [36], and (**x**) proposed method with 0.5 weight factor for clipping operation.

Figure 5 presents experimental results comparing conventional and proposed contrast enhancement methods for visual comparison under the worst driving scenario located in a tunnel. The sequence of methods applied to compute the experimental result frames from Figure 5a–x is the same as in Figure 3. In the worst driving scenario, improving the contrast ratio of the 8-bit input image is crucial for detecting and recognizing objects for autonomous platforms. Enhanced contrast is essential for accurately recognizing the driving status.

Histogram-based contrast enhancement methods exhibit similar trends to the experimental results in the best driving scenario. In Figure 5e,g,i, when MMBEBHE, BPDHE, and BHEPL are applied, the shape of the vehicle in the tunnel is clearly visible, but they do not accurately represent the environment around the vehicle within the tunnel. On the other hand, in Figure 5c,f,h,j, although the clarity in the vehicle region is relatively reduced, the contrast is improved to a level where the driving environment in the tunnel can be roughly judged. However, the overall visual evaluation still feels dark due to low pixel brightness levels observed in Figure 5f,h,j. In Figure 5c, the contrast-enhanced image has a relatively high brightness pixel value compared to Figure 5f,h,j. However, there is a problem of oversaturation in the vehicle region, making it impossible to accurately analyze object characteristics, and there is low contrast enhancement in the background region where the driving environment can be identified. In Figure 5b,d,k,l, the contrast has been improved to the point where the driving environment within the tunnel can be accurately distinguished compared to the results of the other histogram-based contrast enhancement methods. However, the vehicle region is so oversaturated that the wheels and vehicle body cannot be visually distinguished, and the auxiliary lights in the tunnel also appear oversaturated.

In Figure 5x, the proposed method demonstrates a uniform improvement in overall image contrast across all areas. Based on the contrast-enhanced image using our proposed method, it is evident that the pixel brightness level maintains an appropriate value, indicating it is not oversaturated compared to conventional histogram-based enhancement methods. When assessed by regions, the contrast ratio has improved sufficiently to clearly identify the driving environment within the tunnel where the frame was captured. This indicates reasonably good performance among the histogram-based methods.

The experimental results for retinex-based contrast enhancement methods are presented from Figure 5m–s. Among these methods, the SRLLIE [22] method, depicted in Figure 5m, exhibited poor contrast enhancement performance, making it difficult to identify vehicles and the driving environment. However, when utilizing AMSR [18], NPE [19], SIRE [20], SRIE [23], MF [21], and LIME [24], as shown in Figure 5m–r, relatively high contrast enhancement performance was observed. Among these six methods, Figure 5m,o,p, which represents the results of utilizing AMSR [18], SIRE [20], and SRIE [23], respectively, displayed sufficient contrast enhancement in both object and background regions for recognizing the driving environment. However, since the brightness level of the contrast-enhanced images is generally low, post-processing techniques such as gamma correction may be considered to further improve visibility.

The experimental results from Figure 5u–w revealed poor contrast enhancement performance, similar to Figure 5s. However, when employing the method proposed by Dong [25], illustrated in Figure 5t, notable contrast enhancement performance with high edge preservation was observed. Comparing the experimental results from Figure 5r–t, it is important to note that the ranking of user-preferred images may vary based on subjective evaluation. Therefore, a blind test was conducted on the worst driving scenario to rank the images from top to bottom, similar to the methodology described in Table 2 and Figure 4.

**Figure 6:**
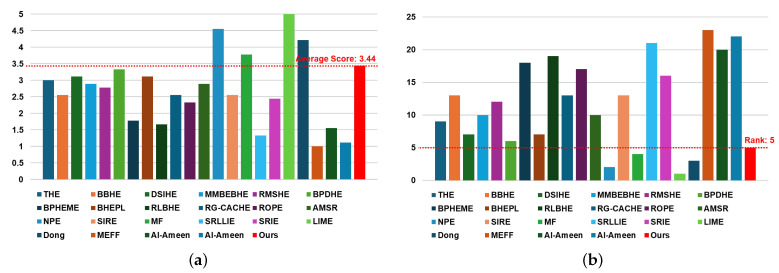
Chart to visually compare Table 3: (**a**) average score and (**b**) rank.

Table 3 and Figure 6 display the subjective evaluation results conducted under blind conditions on a 5-min video, comprising 300 frames depicting the worst driving scenario, similar to the experiments outlined in Table 2. Our method achieved an average score of 3.44 and ranked 5th when sorted from top to bottom. This places our method within the top 30% (approximately within the 7th rank) overall for this scenario. In the histogram-based contrast enhancement method category, our method secured the 1st rank when ranked sequentially, indicating it received the highest average score among its peers. Comparing our proposed method with retinex- or other technique-based contrast enhancement methods, it ranked 5th, placing it within the top 50% (approximately 6 out of 12).

In conclusion, it is evident that no single category of contrast enhancement methods demonstrates overwhelmingly superior performance in the worst driving scenario. A comparison of Table 2 and Table 3 reveals significant performance discrepancies among conventional contrast enhancement methods in subjective visual evaluations across the best and worst driving scenarios. Conversely, our proposed method consistently ranks 6th and 5th in the best and worst driving scenarios, respectively. This consistency offers the advantage of providing users with contrast-enhanced images containing uniform information regardless of the driving conditions.

### 4.2. Quantitative Comparison

In the quantitative comparison, we assess various aspects of contrast enhancement using six metrics: (1) Enhancement Measure (EME), (2) Entropy, (3) Linear Fuzziness (LIF), (4) Lightness Order Error (LOE), (5) Structural Similarity (SS), and (6) Mean Processing Time (MPT). A higher EME value indicates a larger dynamic range within each pre-defined cell, whereas higher values of entropy and SS indicate greater information content in the image. Conversely, lower values of LIF and LOE signify better enhancement.

#### 4.2.1. Best Driving Scenario

**Figure 7:**
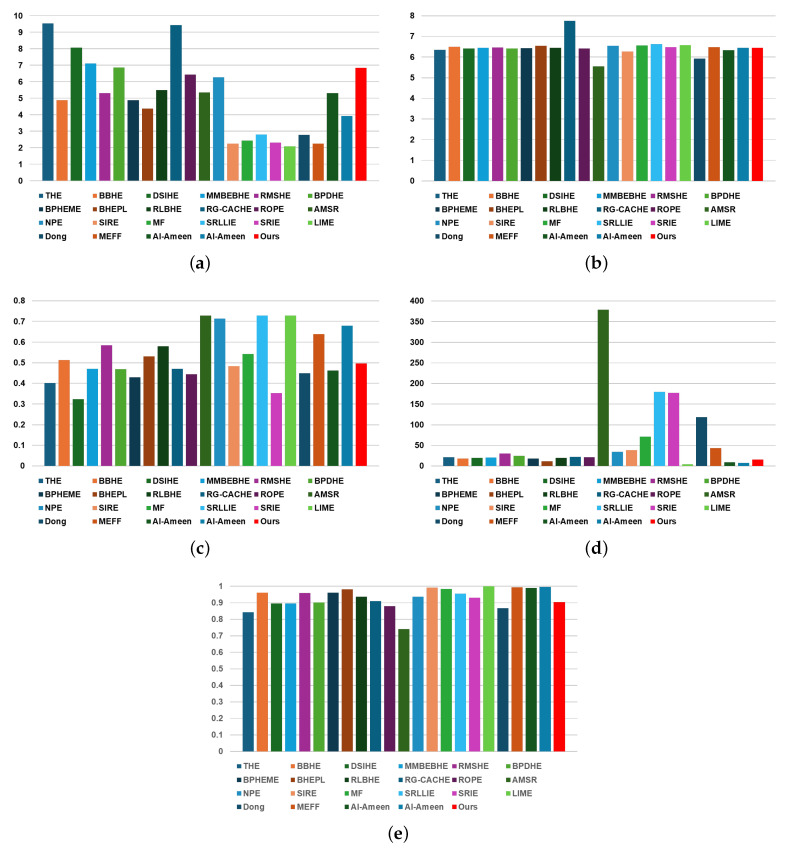
Chart to visually compare Table 4: (**a**) EME, (**b**) Entropy, (**c**) LIF, (**d**) LOE, and (**e**) SS.

Table 4 and Figure 7 present the experimental results of performance evaluation using objective metrics for the best driving scenario frames, as illustrated in Figure 3. When utilizing the proposed method, the EME, entropy, LIF, LOE, and SS metrics are 6.8451, 6.4485, 0.4959, 15.8458, and 0.9043, respectively. For metrics where higher values indicate better performance, the method showing the highest performance in EME is THE with a value of 9.5430, and the proposed method ranked 6th with a value of 6.8451, which means that when considered as a percentage, the proposed method is in the top 30% (approximately within the 7th top rank). When considering Table 2, which shows the subjective evaluation experimental results, it can be seen that the EME values of the proposed method and THE are similar to the average score-based rank results through subjective evaluation.

Regarding entropy, generally, a higher indicator value indicates better performance. In the entropy metric, the proposed method is ranked 12th with a value of 6.4485, indicating moderate performance. In terms of the SS metric, the proposed method is ranked 16th with a value of 0.9043, indicating relatively low performance. However, as shown in Table 2, it can be observed that the entropy and SS values of methods that received good results in subjective evaluation (e.g., THE, RG-CACHE [16], ROPE [17], and the proposed method) are located at low ranks. In other words, in general, as the contrast ratio is greatly improved, it can be said that the better the image quality, the lower the entropy value. This is because the LWIR-based thermal image that can be obtained after the NUC and TC processes basically has a low contrast ratio.

Conversely, for LIF and LOE metrics, a low value indicates high performance. In terms of LIF, when using the proposed method, it ranks 12th with a value of 0.4959, indicating medium performance compared to conventional contrast enhancement methods. Regarding LOE, when using the proposed method, it ranks 5th with a value of 15.8458, indicating high performance (within the top 25%) compared to conventional contrast enhancement methods. The objective performance evaluation results, including LOE and LIF, of the proposed method were satisfactory. However, it is noted that LOE and LIF also exhibit poor index values for conventional methods that received good average scores in subjective evaluation. Therefore, this suggests that the previously used objective indicators cannot be relied upon as a sole standard when evaluating contrast improvement results for LWIR-based thermal images computed after NUC and TC processes in the best driving scenarios.

#### 4.2.2. Worst Driving Scenario

**Figure 8:**
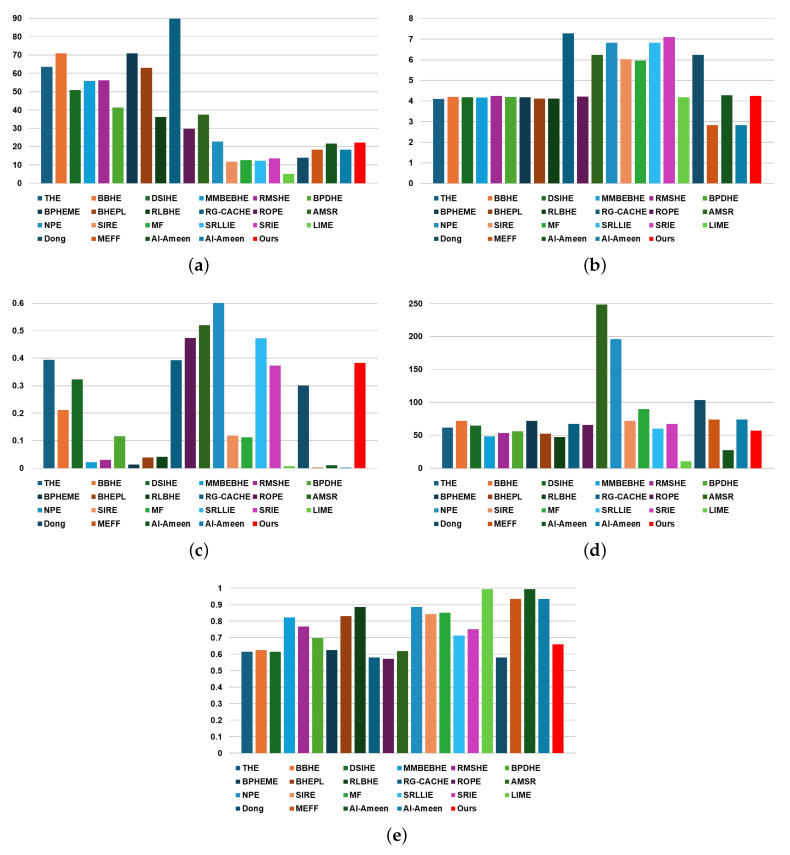
Chart to visually compare Table 5: (**a**) EME, (**b**) Entropy, (**c**) LIF, (**d**) LOE, and (**e**) SS.

Table 5 and Figure 8 showcase the experimental results of performance evaluation for the worst driving scenario frames, as depicted in Figure 5. In terms of EME, the proposed method obtained a rank of 14, from top to bottom, with a value of 22.1756. However, it is essential to note that EME computation relies on the minimum and maximum values per pre-defined cell. Consequently, in worst-case scenarios where the contrast ratio is enhanced, extreme brightness or darkness may skew EME results. Thus, EME may not offer a fair comparison metric as it could be influenced by factors like image illuminance, especially in experimental results of the worst driving scenario using LWIR-based thermal images.

Similarly, the SS metric assumes high structural visibility in the original image. However, in worst-case scenarios, the input image for contrast enhancement lacks clear structure due to an extreme low dynamic range acquired by low infrared energy. Therefore, conversely, a lower SS value might indicate better performance in such scenarios. This is because an image with improved contrast has a specific structure unlike the original input image, making it significantly different. Hence, a smaller SS value indicates better image quality. Based on this understanding, for the SS metric, the proposed method ranked 9th with a value of 0.6606, placing it in the top 40%. Therefore, when using the proposed method for the worst driving scenario, it showed medium performance.

In terms of the LIF, LOE, and entropy metrics, they are calculated using the original image, making them more reliable for understanding the overall driving environment. However, since the original image has low structure and poor dynamic range characteristics, these metrics may not be efficient for objectively evaluating contrast enhancement performance in the worst driving scenario. This is particularly evident when considering the experimental results presented in Table 3 and Table 5.

#### 4.2.3. Processing Speed Performance

**Figure 9:**
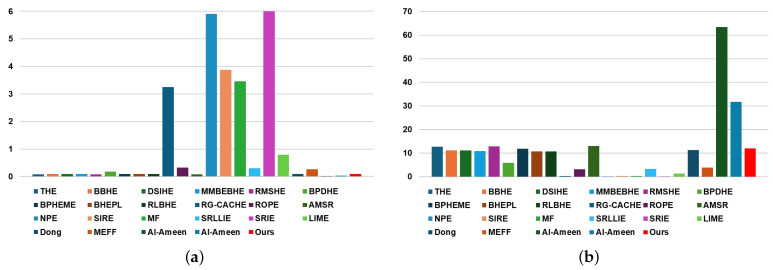
Chart to visually compare Table 6: (**a**) MPT and (**b**) FPS.

Table 6 and Figure 9 present the MPT and frames-per-second (FPS) metrics for both the proposed and conventional methods. These metrics were extracted through experiments conducted using MATLAB software (R2023a version) on a personal computer environment. The MPT values for the proposed and conventional methods were computed based on 200 frames with a resolution of 640 × 480 obtained from the QuantumRed product of Hanwha Systems Company.

Among the histogram-based contrast enhancement methods, except for BPDHE [33], the proposed and conventional methods exhibited similar MPT performance. Converting MPT to FPS yields performance ranging from approximately 10.7 to 12.8 FPS across methods. The proposed method ranked third in terms of both MPT and FPS indicators. However, these values fall short of the real-time performance benchmark of 30 FPS.

Among the retinex-based contrast enhancement methods, NPE [19], SIRE [20], SRIE [23], and SRLLIE [22] required a large amount of processing time, making real-time operation impossible. On the other hand, AMSR [18], MF [21], and LIME [24] required relatively less processing time compared to the other retinex-based contrast enhancement methods. When comparing our proposed method with the retinex-based contrast enhancement methods, our proposed method showed appropriate processing performance.

In terms of the other technique-based contrast enhancement methods, they exhibited fast processing speeds compared to both histogram-based and retinex-based contrast enhancement methods. However, it is evident from previous experimental results that methods such as those proposed by [27,36], which achieve the real-time performance of 30 FPS or higher, exhibit poor contrast enhancement performance in both the best and worst driving scenarios.

In conclusion, methods that demonstrate a certain level of performance in previous experimental results do not achieve real-time processing speeds of more than 30 FPS on a personal computer. Furthermore, there is a risk of performance degradation when running these methods in embedded environments. Solutions for achieving real-time performance will be discussed in Section 5.

## 5. Discussion

### 5.1. Industry Contribution

In Section 4, we visually compared two driving scenarios (best and worst case) and conducted a qualitative evaluation using six metrics. The application of the proposed method demonstrates significant improvements, particularly in terms of enhanced sharpness and contrast ratio in both the best and worst driving scenarios. These findings suggest that the proposed method holds promise for potential use in mass-produced products.

This paper introduces a region-based histogram equalization algorithm with dynamic clipping technique for enhancing N-bit original images following NUC and TC processes, a topic not previously explored in the literature. By combining objective and subjective evaluations, our study provides comprehensive performance evaluation results. We anticipate that our findings will enable companies in defense and electronics industries to implement stable methods for mass-producing products utilizing LWIR-based thermal cameras. However, whereas our proposed method shows promising performance, qualitative evaluation metrics yielded mixed results, indicating the need for further investigation into their alignment with objective/subjective evaluations by actual users.

### 5.2. Contrast Enhancement Performance

**Figure 10:**
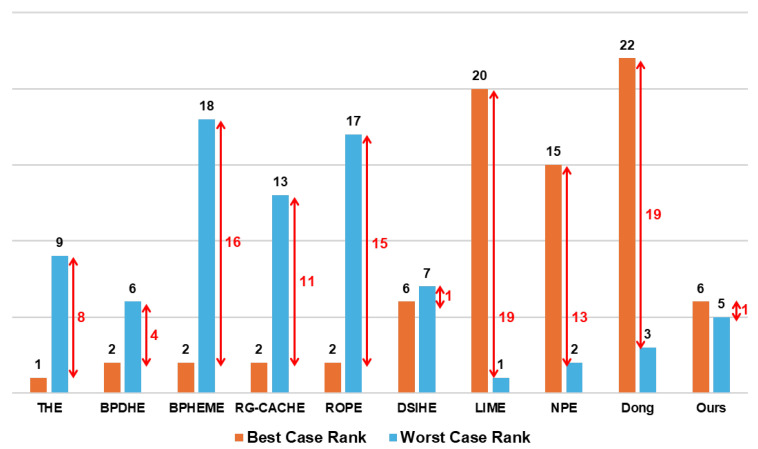
Top rank difference between best and worst driving scenarios.

Figure 10 illustrates the performance disparity between methods that excelled in subjective evaluation in the best and worst driving scenarios. Notably, methods like THE, BPDHE, BPHEME, RG-CACHE, and ROPE, which performed well in the best scenario, exhibit significant rank differences ranging from 4 to 16 or more in the worst scenario. Conversely, LIME, NPE, and the method proposed by Dong, which showed effectiveness in the worst scenario, display rank differences of at least 13 to 19. Interestingly, DSIHE and our proposed method demonstrate minimal variation in ranking between the best and worst scenarios. However, our method consistently ranks higher (6th and 5th) compared to DSIHE (6th and 7th), indicating superior performance and uniform image information provision on average. Therefore, for LWIR-based thermal-imaging cameras used in defense and electronics industries, our proposed method emerges as a viable choice due to its ability to consistently deliver uniform information to users.

### 5.3. Processing Speed Performance with Production Cost

Regarding processing speed performance, neither the proposed method nor conventional methods achieved real-time processing performance in a personal computer environment with a resolution of 640 × 480. The primary limitation arises from executing a contrast enhancement algorithm on a CPU, where only one frame of the input image can be stored in memory at a time, followed by subsequent calculations.

Considering the CPU-based operation mechanism, if all algorithms (including NUC, TC, and contrast enhancement) for thermal image processing are executed in an embedded environment, not only will latency increase, but FPS will also fall short. This deficiency presents a critical challenge as it fails to meet the low latency and processing speed requirement of 30 FPS or higher, typically demanded in the defense and automotive industries. This shortfall is particularly significant because both defense and automotive industries now demand resolutions higher than high definition (HD, 1280 × 720), alongside low latency and ultra-high FPS performance. Specifically, automotive applications necessitate up to 60 FPS for ADAS systems in high-speed driving environments, whereas defense systems require up to 100 FPS to counteract high-speed weapons.

Therefore, considering the pre-processing steps (NUC and TC) before the contrast enhancement algorithm, it becomes imperative to employ a thermal imaging processor equipped with accelerators optimized for these algorithms to meet the processing performance requirements across various resolutions in embedded environments. For the pre-processing accelerator, the RTL (Register Transfer Level) circuit can be designed with a fully pipelined architecture.

In the case of contrast enhancement, encompassing both proposed and conventional algorithms, the RTL circuit may not support a fully pipelined architecture. However, by utilizing internal FIFO memory to store the input frame of the image and simultaneously calculate the histogram, latency, and processing time can be significantly reduced compared to the CPU operation mechanism. If such an optimized accelerator-based thermal imaging processor is applied to the product, the production cost of the final LWIR-based camera can be lowered because only optimized hardware resources (e.g., block random access memories (BRAMs), LUTs, and registers) are used.

## 6. Conclusions

In this paper, we introduced a histogram equalization-based contrast enhancement method employing a region-based clipping technique tailored for dedicated LWIR-based thermal image processing. To assess its performance, we conducted visual and qualitative evaluations comparing the proposed method with conventional approaches under both best and worst driving scenarios. In the visual evaluation, it is evident that the proposed method enhances contrast and clarity compared to the conventional method. In qualitative evaluations of image processing performance and processing speed, the proposed method consistently demonstrates above-average metric results compared to the conventional method in both best and worst driving scenarios. However, as discussed in Section 5, the objective evaluation metrics did not reflect the proposed method’s performance adequately. Hence, future work will involve conducting experiments to gauge the discrepancy between the objective evaluation metrics and user perspectives, with input from a larger pool of test evaluators.

Considering the processing speed, neither the proposed nor conventional methods met real-time performance standards. Therefore, our forthcoming endeavors will concentrate on assessing and improving processing speed. This will entail developing an accelerator in the form of a contrast enhancement processor using field-programmable gate array (FPGA), alongside exploring the development of application-specific integrated circuit (ASIC). Additionally, we will focus on comparing and analyzing the performance of dedicated contrast enhancement processors for infrared-based thermal imaging.

Through the findings presented in this paper and future research experiments, we anticipate significant enhancements in the image quality of mass-produced LWIR-based thermal cameras for night vision systems.

## Figures and Tables

**Table 1:** Various conventional contrast enhancement methods.

Category	Method	Year
	RLBHE [15]	2013
Histogram-based	RG-CACHE [16]	2020
	ROPE [17]	2021
	AMSR [18]	2013
	NPE [19]	2013
	SIRE [20]	2015
Retinex-based	MF [21]	2016
	SRLLIE [22]	2016
	SRIE [23]	2016
	LIME [24]	2017
	Dong [25]	2011
Others	MEFF [26]	2017
	Al-Ameen [27]	2020

**Table 2:** Subjective evaluation of various methods in best driving scenario: results from nine participants on 300 frames.

Method	Person A	Person B	Person C	Person D	Person E	Person F	Person G	Person H	Person I	Average Score	Rank
THE	5	5	5	5	4	5	5	5	5	4.89	1
[29]	4	3	2	3	2	3	3	4	4	3.11	12
[30]	4	5	4	4	5	4	5	5	5	4.56	6
[31]	4	4	4	4	3	4	5	5	5	4.22	8
[32]	4	3	2	4	1	4	4	4	4	3.33	10
[33]	5	5	4	5	5	4	5	5	4	4.67	2
[34]	5	4	5	5	4	4	5	5	5	4.67	2
[35]	4	3	2	3	2	3	4	4	4	3.22	11
[15]	4	3	3	4	3	4	4	4	4	3.67	9
[16]	5	4	5	5	5	3	5	5	5	4.67	2
[17]	5	4	4	5	4	5	5	5	5	4.67	2
Ours	5	4	3	5	4	5	5	5	5	4.56	6
[18]	1	1	1	1	1	1	1	1	1	1.00	22
[19]	3	2	1	2	3	2	3	3	3	2.44	15
[20]	1	1	1	1	2	2	2	2	2	1.56	21
[21]	3	3	2	4	5	2	3	3	3	3.11	12
[22]	2	3	1	2	2	1	3	3	3	2.22	16
[23]	2	2	1	2	4	2	2	2	3	2.22	16
[24]	2	2	1	2	3	1	2	2	2	1.89	20
[25]	1	1	1	1	1	1	1	1	1	1.00	22
[26]	2	2	1	2	2	2	3	3	3	2.22	16
[27]	3	3	1	2	1	1	3	3	3	2.22	16
[36]	2	3	1	3	3	2	3	3	3	2.56	14

**Table 3:** Subjective evaluation of various methods in worst driving scenario: results from nine participants on 300 frames.

Method	Person A	Person B	Person C	Person D	Person E	Person F	Person G	Person H	Person I	Average Score	Rank
THE	4	4	2	2	4	2	2	4	3	3.00	9
[29]	4	3	2	2	3	2	2	3	2	2.56	13
[30]	4	5	2	2	5	2	2	3	3	3.11	7
[31]	3	3	3	3	2	3	3	4	2	2.89	10
[32]	4	2	2	2	3	2	3	4	3	2.78	12
[33]	3	2	3	5	5	3	3	4	2	3.34	6
[34]	3	1	2	1	2	1	2	2	2	1.78	18
[35]	3	3	3	3	4	3	3	4	2	3.11	7
[15]	3	1	2	2	1	1	1	2	2	1.67	19
[16]	4	2	2	2	4	3	2	2	2	2.56	13
[17]	4	2	2	2	3	2	2	2	2	2.33	17
Ours	5	4	2	3	4	3	2	4	4	3.44	5
[18]	5	3	3	3	2	2	2	3	3	2.89	10
[19]	5	5	4	4	5	4	4	5	5	4.56	2
[20]	4	3	2	3	1	2	2	3	3	2.56	13
[21]	4	4	4	4	4	2	4	4	4	3.78	4
[22]	1	2	2	1	1	1	1	2	1	1.33	21
[23]	2	3	2	2	2	1	4	3	3	2.44	16
[24]	5	5	5	5	5	5	5	5	5	5.00	1
[25]	5	4	5	4	3	4	4	4	5	4.22	3
[26]	1	1	1	1	1	1	1	1	1	1.00	23
[27]	1	2	2	1	3	1	1	2	1	1.56	20
[36]	1	1	1	1	2	1	1	1	1	1.11	22

**Table 4:** Performance evaluation for the best driving scenario frame.

Method	EME	Entropy	LIF	LOE	SS
THE	9.5430	6.3503	0.4018	21.3017	0.8432
BBHE [29]	4.8884	6.4911	0.5127	18.0800	0.9601
DSIHE [30]	8.0627	6.4191	0.3231	19.7817	0.8959
MMBEBHE [31]	7.1076	6.4420	0.4699	20.7842	0.8962
RMSHE [32]	5.3176	6.4558	0.5840	30.4000	0.9578
BPDHE [33]	6.8636	6.4213	0.4692	24.2925	0.9018
BPHEME [34]	4.8884	6.4249	0.4302	18.0800	0.9601
BHEPL [35]	4.3751	6.5413	0.5312	11.7825	0.9804
RLBHE [15]	5.4903	6.4454	0.5794	19.8967	0.9368
RG-CACHE [16]	9.4475	7.7473	0.4698	22.0458	0.9101
ROPE [17]	6.4407	6.4118	0.4450	20.8467	0.8787
AMSR [18]	5.3533	5.5537	0.7290	378.7333	0.7407
NPE [19]	6.2717	6.5510	0.7138	34.3925	0.9368
SIRE [20]	2.2524	6.2699	0.4839	38.4483	0.9918
SRIE [23]	2.4411	6.5677	0.5419	71.3442	0.9824
MF [21]	2.8083	6.6197	0.7287	179.5583	0.9543
LIME [24]	2.3124	6.4809	0.3529	177.1883	0.9297
SRLLIE [22]	2.0922	6.5734	0.7285	3.6983	0.9998
Dong [25]	2.7922	5.9300	0.4487	118.7650	0.8662
MEFF [26]	2.2566	6.4797	0.6385	43.1033	0.9945
Al-Ameen [27]	5.3137	6.3279	0.4614	8.9917	0.9903
Al-Ameen [36]	3.9181	6.4510	0.6800	7.4750	0.9965
Ours	6.8451	6.4485	0.4959	15.8458	0.9043

**Table 5:** Performance evaluation for worst driving scenario in a tunnel.

Method	EME	Entropy	LIF	LOE	SS
THE	63.6363	4.0922	0.3943	61.6200	0.6142
BBHE [29]	70.8164	4.1989	0.2120	71.9383	0.6259
DSIHE [30]	50.9569	4.1884	0.3231	65.0767	0.6139
MMBEBHE [31]	55.8149	4.1720	0.0220	48.4775	0.8221
RMSHE [32]	56.1822	4.2422	0.0298	53.7292	0.7669
BPDHE [33]	41.6762	4.1997	0.1157	55.9900	0.6980
BPHEME [34]	70.8164	4.1805	0.0133	71.9383	0.6259
BHEPL [35]	62.9209	4.1103	0.0391	52.8108	0.8314
RLBHE [15]	36.2587	4.1172	0.0416	47.7275	0.8866
RG-CACHE [16]	90.1348	7.2769	0.3934	67.1533	0.5797
ROPE [17]	29.7584	4.2093	0.4740	65.7525	0.5729
AMSR [18]	37.4381	6.2452	0.5197	248.4500	0.6192
NPE [19]	22.7990	6.8297	0.6023	196.2258	0.8866
SIRE [20]	11.8167	6.0200	0.1186	71.9092	0.8443
SRIE [23]	12.6207	5.9563	0.1119	89.6333	0.8521
MF [21]	12.2459	6.8288	0.4729	60.2250	0.7137
LIME [24]	13.5070	7.1112	0.3734	67.6200	0.7514
SRLLIE [22]	5.1925	4.1793	0.0074	10.8800	0.9942
Dong [25]	13.8657	6.2326	0.3013	103.7392	0.5805
MEFF [26]	18.3571	2.8246	0.0020	74.0692	0.9354
Al-Ameen [27]	21.6101	4.2750	0.0107	27.7633	0.9937
Al-Ameen [36]	18.3571	2.8246	0.0020	74.0692	0.9354
Ours	22.1756	4.2490	0.3829	56.9617	0.6606

**Table 6:** Mean processing time (MPT) and frame-per-second (FPS) performance.

Category	Method	Mean Processing Time (s)	Frame-Per-Second (FPS)
	THE	0.07830	12.7714
	BBHE [29]	0.08980	11.1359
	DSIHE [30]	0.08945	11.1794
	MMBEBHE [31]	0.09155	10.9230
	RMSHE [32]	0.07815	12.7959
Histogram-based	BPDHE [33]	0.16955	5.8980
	BPHEME [34]	0.08425	11.8694
	BHEPL [35]	0.09345	10.7009
	RLBHE [15]	0.09325	10.7239
	RG-CACHE [16]	3.24542	0.3081
	ROPE [17]	0.31558	3.1688
	Ours	0.08340	11.9904
	AMSR [18]	0.07689	13.0056
	NPE [19]	5.90948	0.1692
	SIRE [20]	3.87817	0.2579
Retinex-based	SRIE [23]	3.45799	0.2892
	MF [21]	0.30080	3.3245
	SRLLIE [22]	6.03222	0.1658
	LIME [24]	0.78934	1.2669
	Dong [25]	0.08868	11.2765
Others	MEFF [26]	0.25854	3.8679
	Al-Ameen [27]	0.01577	63.4115
	Al-Ameen [36]	0.03149	31.7561

## Data Availability

Data sharing is not applicable due to data security legal policy related to the Korean military industry.

## References

[1] Khamsehashari R., Schill K. (2021). Improving deep multi-modal 3D object detection for autonomous driving. Proceedings of the 2021 7th International Conference on Automation, Robotics and Applications (ICARA).

[2] Cai Y., Luan T., Gao H., Wang H., Chen L., Li Y., Sotelo M.A., Li Z. (2021). YOLOv4-5D: An effective and efficient object detector for autonomous driving. IEEE Trans. Instrum. Meas..

[3] Patel H., Upla K.P. (2020). Night vision surveillance: Object detection using thermal and visible images. Proceedings of the 2020 International Conference for Emerging Technology (INCET).

[4] Danaci K.I., Akagunduz E. (2024). A survey on infrared image & video sets. Multimed. Tools Appl..

[5] Chen B., Chen Y., Deng Z. (2021). Recent advances in high speed photodetectors for eSWIR/MWIR/LWIR applications. Photonics.

[6] Pinchon N., Cassignol O., Nicolas A., Bernardin F., Leduc P., Tarel J.P., Brémond R., Bercier E., Brunet J. (2019). All-weather vision for automotive safety: Which spectral band?. Proceedings of the Advanced Microsystems for Automotive Applications 2018: Smart Systems for Clean, Safe and Shared Road Vehicles 22nd.

[7] (2018). Road Vehicles Functional Safety.

[8] Dai X., Yuan X., Wei X. (2021). TIRNet: Object detection in thermal infrared images for autonomous driving. Appl. Intell..

[9] Brehar R.D., Muresan M.P., Mariţa T., Vancea C.C., Negru M., Nedevschi S. (2021). Pedestrian street-cross action recognition in monocular far infrared sequences. IEEE Access.

[10] Jiang C., Han J.J. (2022). A Multiobject Detection Scheme Based on Deep Learning for Infrared Images. IEEE Access.

[11] Yadav P.K., Yadav I., Ajitha B., Rajasekar A., Gupta S., Reddy Y.A.K. (2022). Advancements of uncooled infrared microbolometer materials: A review. Sens. Actuators A Phys..

[12] Deane S., Avdelidis N.P., Ibarra-Castanedo C., Zhang H., Yazdani Nezhad H., Williamson A.A., Mackley T., Maldague X., Tsourdos A., Nooralishahi P. (2020). Comparison of cooled and uncooled ir sensors by means of signal-to-noise ratio for ndt diagnostics of aerospace grade composites. Sensors.

[13] Yuan P., Tan Z., Zhang X., Wang M., Jin W., Li L., Su B. (2023). Fixed-pattern noise model for filters in uncooled infrared focal plane array imaging optical paths. Infrared Phys. Technol..

[14] Cao X., Zhu B., Wang S., Yong Y., Huo Y., Huo J. (2019). Nonuniformity Correction Algorithm for TEC-less Uncooled Infrared Imaging System. Proceedings of the 2019 IEEE 5th International Conference on Computer and Communications (ICCC).

[15] Zuo C., Chen Q., Sui X. (2013). Range limited bi-histogram equalization for image contrast enhancement. Optik.

[16] Wu X., Kawanishi T., Kashino K. (2020). Reflectance-guided, contrast-accumulated histogram equalization. Proceedings of the ICASSP 2020—2020 IEEE International Conference on Acoustics, Speech and Signal Processing (ICASSP).

[17] Wu X., Sun Y., Kimura A., Kashino K. (2021). Reflectance-oriented probabilistic equalization for image enhancement. Proceedings of the ICASSP 2021—2021 IEEE International Conference on Acoustics, Speech and Signal Processing (ICASSP).

[18] Lee C.H., Shih J.L., Lien C.C., Han C.C. (2013). Adaptive multiscale retinex for image contrast enhancement. Proceedings of the Signal-Image Technology & Internet-Based Systems (SITIS), 2013 International Conference on Signal-Image Technology & Internet-Based Systems.

[19] Wang S., Zheng J., Hu H.M., Li B. (2013). Naturalness preserved enhancement algorithm for non-uniform illumination images. IEEE Trans. Image Process..

[20] Fu X., Liao Y., Zeng D., Huang Y., Zhang X.P., Ding X. (2015). A probabilistic method for image enhancement with simultaneous illumination and reflectance estimation. IEEE Trans. Image Process..

[21] Fu X., Zeng D., Huang Y., Liao Y., Ding X., Paisley J. (2016). A fusion-based enhancing method for weakly illuminated images. Signal Process..

[22] Li M., Liu J., Yang W., Sun X., Guo Z. (2018). Structure-revealing low-light image enhancement via robust retinex model. IEEE Trans. Image Process..

[23] Fu X., Zeng D., Huang Y., Zhang X.P., Ding X. A weighted variational model for simultaneous reflectance and illumination estimation. Proceedings of the IEEE Conference on Computer Vision and Pattern Recognition.

[24] Guo X., Li Y., Ling H. (2016). LIME: Low-light image enhancement via illumination map estimation. IEEE Trans. Image Process..

[25] Dong X., Wang G., Pang Y., Li W., Wen J., Meng W., Lu Y. (2011). Fast efficient algorithm for enhancement of low lighting video. Proceedings of the 2011 IEEE International Conference on Multimedia and Expo.

[26] Ying Z., Li G., Gao W. (2017). A bio-inspired multi-exposure fusion framework for low-light image enhancement. arXiv.

[27] Al-Ameen Z. (2020). Contrast enhancement of medical images using statistical methods with image processing concepts. Proceedings of the 2020 6th International Engineering Conference “Sustainable Technology and Development” (IEC).

[28] Zuiderveld K. (1994). Contrast limited adaptive histogram equalization. Graphics Gems.

[29] Kim Y.T. (1997). Contrast enhancement using brightness preserving bi-histogram equalization. IEEE Trans. Consum. Electron..

[30] Wang Y., Chen Q., Zhang B. (1999). Image enhancement based on equal area dualistic sub-image histogram equalization method. IEEE Trans. Consum. Electron..

[31] Chen S.D., Ramli A.R. (2003). Minimum mean brightness error bi-histogram equalization in contrast enhancement. IEEE Trans. Consum. Electron..

[32] Chen S.D., Ramli A.R. (2003). Contrast enhancement using recursive mean-separate histogram equalization for scalable brightness preservation. IEEE Trans. Consum. Electron..

[33] Ibrahim H., Kong N.S.P. (2007). Brightness preserving dynamic histogram equalization for image contrast enhancement. IEEE Trans. Consum. Electron..

[34] Wang C., Ye Z. (2005). Brightness preserving histogram equalization with maximum entropy: A variational perspective. IEEE Trans. Consum. Electron..

[35] Ooi C.H., Kong N.S.P., Ibrahim H. (2009). Bi-histogram equalization with a plateau limit for digital image enhancement. IEEE Trans. Consum. Electron..

[36] Al-Ameen Z. (2020). Improving the contrast of aerial images using a new multi-concept algorithm. IEIE Trans. Smart Process. Comput..

